# Value‐based care in surgery: implications in crisis and beyond

**DOI:** 10.1111/ans.17501

**Published:** 2022-04-18

**Authors:** Payal Mukherjee, Mohamed Khadra, Neil Merrett, Ellen Rawstron, Arthur Richardson, Kim Sutherland, Jean‐Frederic Levesque

**Affiliations:** ^1^ Institute of Academic Surgery Royal Prince Alfred Hospital Sydney New South Wales Australia; ^2^ Discipline of Surgery University of Sydney Sydney New South Wales Australia; ^3^ Department of Surgery Nepean Hospital Sydney New South Wales Australia; ^4^ Discipline of Surgery, School of Medicine Western Sydney University Sydney New South Wales Australia; ^5^ Agency for Clinical Innovation NSW Health Sydney New South Wales Australia; ^6^ Department of Surgery Westmead Hospital Sydney New South Wales Australia; ^7^ Centre for Primary Health Care and Equity University of New South Wales Sydney New South Wales Australia

## Introduction

Over the past 2 years, surgeons and surgical systems have demonstrated an ability to rapidly adopt value‐driven care, triage patients and make evidence‐based decisions in response to crisis. Building on these successes, this paper explores a framework to expand these advances in creating a value‐based approach to patient care through clinician leadership and state‐wide clinical networks.

Value‐driven care is often mistaken for cost reduction. While cost considerations are part of the framework, avoidance of low‐value care is multifaceted – derived from improving health outcomes and patient experiences as well as efficient and sustainable deployment of healthcare resources.^1^ When considering the value of care, broad constructs such as appropriateness, effectiveness, safety, efficiency and opportunity cost must be considered.^2^

In Australia, patients access inpatient and outpatient services, using a mixture of private and public resources. Value may be enhanced at various stages of the care delivery process. Thus, assessment of low‐value care should include a whole of system assessment of the patient's journey, ensuring that the right care is provided in the right way using the right resources. This includes assessing *p*atient's needs, *p*roficiency of the system, *p*rocess support, *p*rocedure choice and *p*rocurement efficiency (Table 1).

### Providing the right care

If *patient*'*s needs* are not being met through delivery of care, it is of low value.^2^, ^3^ While clinical guidelines are set by clinicians, clinical variance in rates or outcomes of surgery may flag inequitable access to care or low‐value surgery. Whether variation creates low value or adds to value depends on whether that variation is warranted.^4^ Clinicians are often unaware of such trends, while policy makers may not have the clinical information or expertise required to understand the variance. Unwarranted variation and cost may be reduced by simply implementing existing clinical guidelines where the evidence suggests a procedure is of low value to patients. Conversely, clinical input to understand if variation is warranted may attract additional resources to support care models, such as outreach work to support indigenous health care.

### Providing care in the right way

Low‐value care, from a *process* perspective, considers whether length of stay and pre‐ and post‐operative care are optimized. Surgical outcomes rely not only on the expertise of the surgeon, but also on the team and facility demonstrated by outcomes of high‐volume centres.^5^ When evaluating whether variation is linked to process, resource or outcome, process‐related variation contributes to 73% of the reported literature.^4^ Malik *et al*.^3^ demonstrated that the impact of healthcare resources are not only through high‐cost, high‐volume surgery. Low‐cost, high‐volume surgery also consumes significant resources. Therefore, appropriate care models are important for all types of surgery such as dedicated day‐only facilities for high‐volume cases.

Avoiding low‐value care due to elements of safety and efficacy of care delivery requires optimizing *proficiency*. While morbidity, mortality assessment and surgical audit are core to continuous professional development of each surgeon, very little work is done as a network, so hospitals learn from each other's errors. Variance data of outcomes between institutions and clinicians are held by Government and the Health Insurance sector. Allowing individual clinicians or institutions to benchmark their clinical outcomes and system efficiency across pooled state data could be a powerful force for change. Multidisciplinary collaboration is important in improving proficiency especially in addressing non‐beneficial surgery such as demonstrated by the Agency for Clinical Innovation's Frailty taskforce which seeks input from anaesthetists, geriatricians as well as surgeons for high‐risk surgery with difficult ethical challenges. Rapid introduction of new technologies in surgery introduces challenges in governance. Training is often left to industry with potential conflicts of interest. However, improving institution‐based credentialling^6^ and ongoing evaluation and governance may assist in capturing low‐value care, especially when new technology is adopted by a larger workforce or for new indications not originally intended. Collaboration between public and private sectors and between clinicians and policy makers could be pivotal in developing better system governance in safe and effective deployment of surgical innovation.

### Providing care using the right resources

*Procedure* technique and resources used can also impact low‐value care.^1^ Innovations in surgery may result in reduced length of stay or operating time with similar or improved outcomes. Conversely, it may add to resource utilization (both human and financial).^6^ Value‐based implementation of new procedures should capture outcomes by surgeons as well as by patients. Surgical academics must be proactive in evaluating health economics,^6^ factor direct and indirect costs (such as cost of staff safety, ergonomics and generation of non‐reimbursed work) and opportunity cost of finite resources in the translational research pipeline. These must also be factored into future modelling of hospital resource utilization. For sustainable implementation of new innovations, it is essential to balance the cost and benefit of any new technology, rather than a direct move to a more expensive technique with no proven benefit to the patient as it holds low benefit for the clinician, patient or health service.

### Procurement

*Procurement* costs can vary significantly in our health system. Surgeons may add to this by unconsciously promoting the use of consumables that do not influence outcome but vary greatly in cost. Cost variations and impact on value can reduce system flexibility in a crisis. For example, higher cost of prosthesis procurement by private hospitals may impact the sustainability of private–public partnerships to overcome elective surgery backlog, if private hospitals cannot deliver care at the same cost as public hospitals. Harnessing the state‐wide economies of scale, while slightly reducing choice of prosthesis, can maintain positive patient outcomes as well as reduce resource wastage.

**Table 1:** Potential ways to identify areas of low‐value care in the health system and mechanism to address them

Assess value area	Explanation	Current paradigm	Mechanism for change
Patient needs	Does the patient need surgery	Clinical guidelines set by workforce	(1) Compliance with clinical guidelines (2) Reduce clinical variance (3) Shared decision‐making (4) Quality of life based planning of surgery
Proficiency	Technical competence and reduction of errors	(1) Training in new technologies often driven by industry (2) Credentialling is not systemically ensured for new interventions, especially of new techniques if technology is of low cost	(1) Better governance of credentialling and prospective monitoring of outcomes (in public and private sectors) (2) Obtain MDT input into new procedures or high‐risk care (3) Improve MDT compliance where value is already proven (cancer care)
Process	Improve system efficiency Availability of the entire pre‐ and post‐operative care pathway in a streamlined manner	Hospital and administration driven	(1) Better clinical input and collaboration with administrators and policy makers to improve system efficiency (2) Reduce process‐based variation across institutions (3) Implement appropriate value‐driven care models for high‐ and low‐volume surgery
Procedure	Is the patient getting the most appropriate technique	Once the new technology is introduced, its scale and adoption in other areas of surgery (where evidence maybe limited) is not rigorously monitored. The assessment of value (not just evidence) is not common	(1) Specialty‐specific databases and audits of surgical outcomes (2) Early introduction of prospective databases to measure health economics of new technology (3) Measurement of patient‐reported outcomes and experiences
Procurement	Cost of consumables	High variability within and between different institutions in both public and private sectors	(1) Educate workforce of implications of their choice on cost and climate (2) Attain more transparent costs from industry reducing procurement variability between different institutions

MDT, multidisciplinary.

### Conclusion

Surgeons in Australia demonstrate a high standard of surgical training and professionalism and have a propensity to be drivers of change. In addition, with prolonged stress on the health system and the workforce, surgeons need to be equipped with diverse skills that allow them to adapt to the changing pressures. Surgical culture needs to change to embrace education in financial literacy and health services and to continually challenge their current practices. Partnerships between clinicians and policy makers are key to ensure accurate analysis of, and agile response to, healthcare variation. This will allow better crisis response while maintaining safe, sustainable and equitable healthcare delivery at other times.

### Author contributions

**Mohamed Khadra:** Writing – review and editing. **Neil Merrett:** Writing – review and editing. **Ellen Rawstron:** Writing – review and editing. **Arthur Richardson:** Writing – review and editing. **Jean‐Frederic Levesque:** Writing – review and editing. **Payal Mukherjee:** Writing – original draft.
